# Natural Treg and role of IL-2 in lupus

**DOI:** 10.1186/ar3411

**Published:** 2011-09-16

**Authors:** Gabriela Riemekasten, Jens Y Humrich

**Affiliations:** 1 Department of Rheumatology and Clinical Immunology, Charité University hospital, Berlin, 10117, Germany

## Background

Effector T cells play an important role in the pathogenesis of lupus. As recently shown in murine lupus, they contribute to tissue damage and glomerulonephritis.

## Methods

The role of naturally occurring regulatory T cells (Treg) and of IL-2 was studied in vitro and in vivo by using flow cytometry and the NZB/W lupus mouse model.

## Results

In healthy individuals as well as in young lupus prone mice without any signs of the disease, effector T cells are tightly controlled by naturally occurring regulatory T cells (Treg) that can be shown by different approaches: 1. After depletion of Treg cells by anti-CD25 therapy, murine lupus is strongly accelerated. 2. After passive transfer of Treg (CD4+CD25+ T cells consisting of 95% FoxP3+ T cells), murine lupus improved reflected by reduced proteinuria and increased survival compared to control mice [1]. 3. In vitro depletion of Treg lead to better detection of autoantigen-specific effector T cells with frequencies above the detection limit for flow cytometry. The frequency of autoangien-specific T cells correlate with the disease activity in human and murine lupus. The control of effector T cells by Treg cells can be also used for studying the phenotype of effector T cell and their function at an autoantigen-specific level. However; as shown in the murine NZB/W lupus model, there is a progressive loss of Treg/Tcon homeostasis during lupus development in different compartments with a progressive Treg deficiency. In lupus mice with proteinuria, the phenotype of effector T cells is very similar to the T cell phenotype obtained in IL-2 deficient mice. As known from the literature, IL-2 levels are decreased in SLE patients. According to the characteristics of Treg, they are more sensitive to IL-2 deficiency. Supporting this, addition of IL-2 resulted in a dominant proliferation of Treg cells. In murine lupus, IL-2 improved survival and decreased proteinuria in diseased NZB/W mice.

## Conclusions

Our data support the possible role of Treg and of IL-2 supplementation in lupus therapy. Further studies are underway to evaluate IL-2 supplementation and its effects on immune cells and disease symptoms in lupus.
